# Acquired renal glucosuria in an undifferentiated connective tissue disease patient with a *SLC5A2* heterozygous mutation: A case report

**DOI:** 10.1097/MD.0000000000013664

**Published:** 2018-12-14

**Authors:** Xiangchen Gu, Min Chen, Yanqiu Xu, Yi Wang

**Affiliations:** Department of Nephrology, Yueyang hospital of Integrated Traditional Chinese and Western Medicine, Shanghai University of TCM, China.

**Keywords:** renal glucosuria, SGLT2, *SLC5A2*, UCTD

## Abstract

**Introduction:**

Renal glucosuria is a renal tubular disorder caused by genetic conditions, drugs, and poisons. Mutations in the *SLC5A2* gene are recently found to be responsible for the inherited renal glucosuria, while undifferentiated connective tissue disease (UCTD) was not considered pathogenic for renal glucosuria. Here, we present a case of acquired renal glucosuria in a UCTD patient.

**Patient concerns:**

A 30-year-old woman was seen in the outpatient clinic for complaints of frequent urination and dysuria. Laboratory tests showed a urinary tract infection (UTI) and persistent renal glucosuria. After antibiotic treatment, the UTI symptoms were relieved, but the renal glucosuria remained.

**Diagnosis:**

Laboratory tests ruled out renal tubular acidosis and diabetes mellitus. Genetic analysis showed a heterozygous mutations in the *SLC5A2* gene. Meanwhile, immunological tests showed a high antinuclear antibody titer (1:160) and an elevated anti-Rho/SSA antibody level. Schirmer test, tear breakup time, and lip biopsy results were all negative. The patient did not meet the criteria for any known connective diseases. Therefore, she was diagnosed with UCTD. Interventions: The patient was started with the treatment of Hydroxychloroquine.

**Outcomes:**

Hydroxychloroquine treatment resolved the renal glucosuria. The patient's follow- up urinalysis showed no glucosuria at all.

**Lessons:**

This is the first case report to demonstrate that UCTD may induce renal glucosuria in a patient with a heterozygous mutation in *SLC5A2*. This case suggests that during the process of diagnosing renal glucosuria, in addition to familial renal glucosuria (FRG), autoimmune diseases, though rare, should also be taken into consideration.

## 1 Introduction

Renal glucosuria is a renal tubular disorder characterized by persistent isolated glucosuria in the absence of hyperglycemia.^[1]^ The causes of renal glucosuria include a wide range of insults to proximal tubular cells, including genetic conditions, drugs, and poisons.^[2]^ Mostly, the inherited form of this disorder is called familial renal glucosuria (FRG), which is a rare renal tubular disorder due to autosomal recessive mutations in the *SLC5A2* gene located on human chromosome 16p11.2.^[3,4]^ The *SLC5A2* gene encodes the low-affinity sodium/glucose cotransporter SGLT2, which is responsible for most glucose reabsorption in the kidney.^[5,6]^ A previous study has suggested that overt glucosuria requires the individual to be homozygous or compound heterozygous.^[7]^

Herein, we report an unusual case of isolated renal glucosuria in an undifferentiated connective tissue disease (UCTD) patient with a heterozygous mutation in *SLC5A2*.

## 2 Case presentation

A 30-year-old Chinese woman was referred to the renal division because of frequent urination and dysuria for almost 2 weeks. She had been healthy without any diagnosed diseases, she was not taking any medications, and her clinical examination revealed no significant findings.

**Figure 1:**
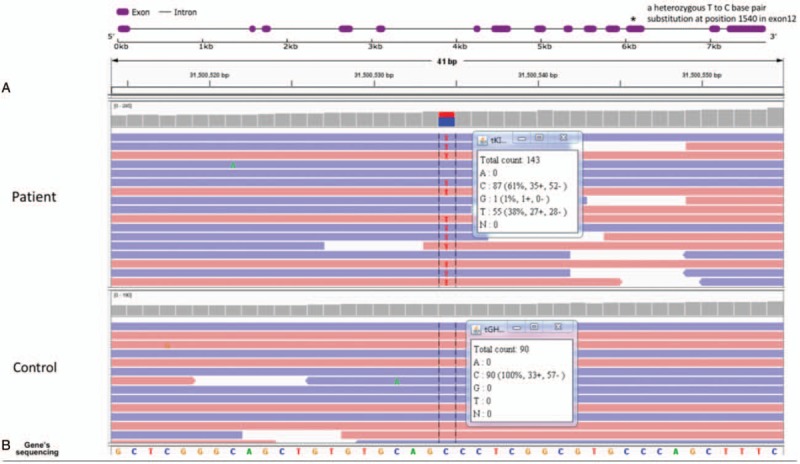
Genome sequencing of all exon regions of *SLC5A2* (A) Schematic diagram of the *SLC5A2* gene, with exons (boxes) and introns (lines). (B) Genome sequencing revealed a heterozygous mutation in C1540T which causes Pro to Ser substation at position 1540.

The laboratory results showed pyuria and bacteriuria, which suggested a clinical diagnosis of urinary tract infection (UTI). The oral antibiotic levofloxacin was used to treat the UTI. After the treatment, the urinalysis was normal except for persistent glucosuria (urine glucose +-2+). Other test results were as follows: fasting plasma glucose (4.74 mmol/L), hemoglobin A1C 5.1%, oral glucose tolerance test (5.4 mmol/L), urine microalbumin/creatinine (9.89 mg/g), serum potassium (4.6 mmol/L), serum sodium (139 mmol/L), chloride (97 mmol/L), and bicarbonate (23.7 mmol/L). Diabetes mellitus, renal tubular acidosis, and Fanconi syndrome were excluded. Then, FRG was taken into consideration. With the consent of the patient, genomic DNA was extracted from peripheral leukocytes. The entire coding region and adjacent intronic segments of *SLC5A2* were screened for mutations by genome sequencing (Jinyu, Shanghai). The results showed that the patient had a heterozygous C to A base pair substitution at 1540 in exon 12. This mutation causes a Pro to Ser missense mutation at position 514 (Fig. 1). The heterozygous mutation has not been reported before and may not cause mild to moderate glucosuria. To rule out other possible diseases, immunological tests were performed. Surprisingly, the tests showed a high antinuclear antibody (ANA) titer (1:160), an elevated anti-Rho/SSA antibody level, a decrease in C3 and C4, and normal IgG, IgA, IgM, and RF levels. Schirmer test, tear breakup time, and lip biopsy results were all negative (Table 1). The patient did not meet the criteria for any known connective diseases. Therefore, she was diagnosed with UCTD. The patient was then treated with Plaquenil (hydroxychloroquine) 100 mg twice a day orally for 6 months. The patient's monthly follow-up urinalysis showed weakly positive to negative glucosuria during 6 months of treatment (Table 2).

**Table 1:**
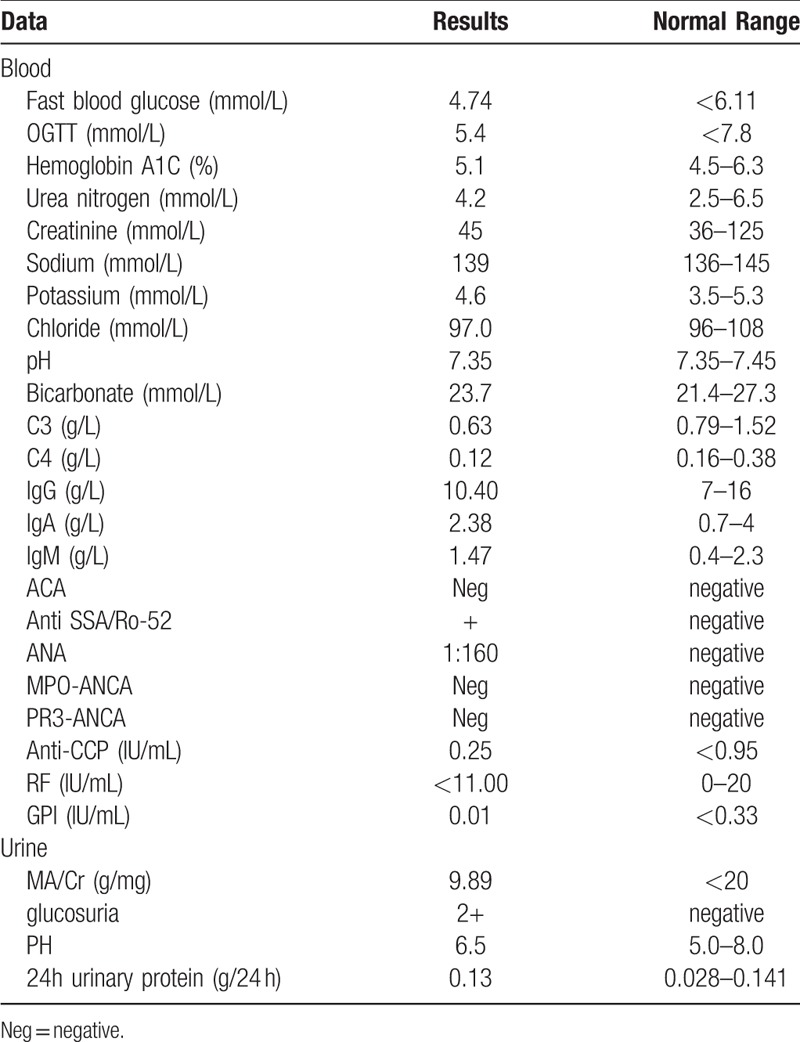
Laboratory results of the patient before treatment.

**Table 2:**
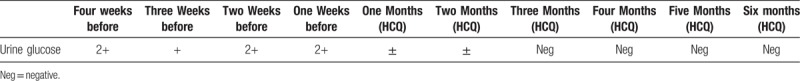
Urine glucose before and with hydroxychloroquine (HCQ) treatment.

## 3 Discussion

Under normal physiological conditions, proximal tubules in the kidneys reabsorb almost 180 g of glucose daily via SGLT2, a kidney-specific transporter, accounting for the reabsorption of glucose filtered by glomeruli.^[8]^ The protein SGLT2, encoded by *SLC5A2*, a 7.6 kb gene containing 14 exons, has 672 amino acids, with an inferred secondary structure consisting of 14 transmembrane-spanning domains and both the NH2 and COOH termini facing the extracellular milieu.^[9]^

Thus, mutations in the *SLC5A2* gene are responsible for renal glucosuria. A previous study has suggested that homozygous or compound heterozygous mutations in the *SLC5A2* gene can cause overt glucosuria.^[7]^ Other research has also indicated that the heterozygous mutation of *SLC5A2* might cause “mild” glucosuria.^[10]^ However, increased glucose excretion was not observed in all individuals with similar or identical mutations, heterozygosity, in particular, indicating that other genetic or nongenetic factors are involved.^[11]^

In this patient, nongenetic factors were also considered because she only had 1 heterozygous mutation in *SLC5A2* in exon 12 and had never presented with glucosuria before. Surprisingly, the immunological tests demonstrated a high ANA titer (1:160) and positive antibodies to Rho/SSA. The serological profile in this patient was consistent with Sjögren syndrome (SS). However, she did not have any other associated symptoms suggestive of primary SS (pSS); thus, the patient was diagnosed with UCTD.^[12]^

The term undifferentiated connective tissue disease (UCTD) refers to unclassifiable systemic autoimmune disorders that share clinical and serological manifestations with definite connective tissue diseases (CTDs), such as systemic lupus erythematosus (SLE), systemic sclerosis (SSc), SS, dermatomyositis/polymyositis (DM/PM), mixed connective tissue disease (MCTD) and rheumatoid arthritis (RA), but not fulfilling any of the existing classification criteria.^[13]^ This disease most commonly presents symptoms such as arthralgia, arthritis, Raynaud phenomenon, leukopenia, xerostomia, and xerophthalmia.^[14]^ In some rare cases, UCTD may present as cardiac tamponade, fatal cardiac arrest, membranoproliferative glomerulonephritis (MPGN), and podocyte infolding glomerulopathy (PIG),^[15–18]^ indicating that UCTD induces the autoantibody-mediated immune response, leading to different types of damage to different tissues, including tissues in the cardiovascular and renal systems.

Meanwhile, the presence of anti-SSA antibodies in both the 52 and 60 kD antigens has been correlated with progression to pSS.^[19]^ In one UCTD patient cohort, those with anti-SSA and/or anti-SSB antibodies presented an SS-like disease.^[19,20]^ In this patient, a high ANA titer and positive antibodies to Rho/SSA indicate progression to pSS, a well-defined CTD, in the future.

Furthermore, this patient has a heterozygous mutation in *SLC5A2* in exon 12. According to the postulated topology of SGLTs, it has been suggested that transmembrane domains 10 to 13 may play a pivotal role in sugar binding and translocation.^[7,9,21]^ Therefore, it is reasonable to assume that mutations in these domains would result in significantly impaired SGLT2 function, leading to severe glucosuria. Indeed, Santer et al reported cases presenting with extreme glucosuria (glucose excretion >100 g/1.73 m^2^/day), with either homozygous or double heterozygous mutations affecting SGLT2 residues of transmembrane domains 10 to 13.^[7]^

Thus, the heterozygous mutation in exon 12 could be responsible for latent SGLT2 hypofunction, and the underlying UCTD-associated autoimmunity could have induced the manifestation of its hypofunction. Therefore, the autoantibody to SGLT2 may be a nongenetic factor of the mild to moderate glucosuria in this patient with a heterozygous mutation in *SLC5A2*.

Interestingly, the same condition has been reported in other cases. An anti-SSA antibody-positive patient was diagnosed with acquired Gitelman syndrome with an *SLC12A3* heterozygous mutation. The researcher presumed that the heterozygous mutation could be responsible for the latent hypofunction of NCCT causing acquired Gitelman syndrome.^[22]^ We also reported the case of a pSS patient with an *SLC12A3* heterozygous mutation presenting with acquired Gitelman syndrome.^[23]^ Therefore, all of these case reports indicate that patients with autoimmune diseases, especially those on the spectrum of SS-like diseases, are more susceptible to renal tubular disorders, typically patients with heterozygous mutations in genes encoding renal tubular transporters.

Additionally, renal glucosuria fully recovered after the treatment of UCTD with hydroxychloroquine; this finding strongly supports the mediation of renal glucosuria by autoantibodies. Data have shown that in SLE, early intervention with hydroxychloroquine can delay or prevent the development of more severe sequelae.^[24]^ At the same time, it is important to follow up with this patient regularly, as her condition might eventually evolve into pSS.

This case report has some limitations. First, glucosuria was not quantitatively examined but was only qualitatively expressed as positive. Additionally, in general, renal biopsies are unnecessary for isolated renal glucosuria patients, so we did not confirm the hypothesis in this case report by a kidney biopsy. Thus, it is impossible for us to obtain histological evidence of the direct autoantibody anti-SGLT2 in proximal tubular epithelial cells. Unfortunately, we could not perform immunofluorescence or immunoblot analysis to test for the presence of autoantibodies reactive to kidney tissue or endogenous kidney protein.

To the best of our knowledge, this is the first report to demonstrate that UCTD-associated autoimmunity may induce renal glucosuria in a patient with an *SLC5A2* heterozygous mutation. This case suggests that during the process of diagnosing renal glucosuria, in addition to FRG, autoimmune diseases, though rare, should also be taken into consideration.

## Acknowledgments

Thank you.

## Author contributions

**Formal analysis:** Xiangchen Gu, Yanqiu Xu.

**Investigation:** Xiangchen Gu, Min Chen.

**Project administration:** Yanqiu Xu.

**Supervision:** Min Chen, Yi Wang.

**Writing – original draft:** Xiangchen Gu.

**Writing – review & editing:** Yi Wang.
